# Exosomes in oral squamous cell carcinoma: functions, challenges, and potential applications

**DOI:** 10.3389/fonc.2024.1502283

**Published:** 2025-01-16

**Authors:** Bo Zhao, Zuntai Li, Ronghua Li

**Affiliations:** ^1^ Key Laboratory of Advanced Intelligent Protective Equipment Technology (Hebei University of Technology), Ministry of Education, Tianjin, China; ^2^ Department of Stomatology, Tianjin First Central Hospital, Tianjin, China

**Keywords:** extracellular vesicles, biological characteristics, OSCC, oncology, exosomes

## Abstract

Oral squamous cell carcinoma (OSCC) accounts for approximately 90% of all oral cancers, significantly impacting the survival and quality of life of patients. Exosomes, small extracellular vesicles released by cells, play a crucial role in intercellular communication in cancer. Nevertheless, their function and mechanism in OSCC remain elusive. Search Pubmed, Web of Science, and Cochrane Library using keywords OSCC, exome, diagnosis, and treatment to review the research progress of exome in OSCC. Based on these results, this review starting from the biosynthesis, structure, and contents of exosomes, elaborates on the research progress of exosomes in the diagnosis and treatment of OSCC. It explores the potential of exosomes in the diagnosis and treatment of OSCC, and briefly describes the challenges researchers currently face.

## 1 Introduction

Exosomes represent one of the most prevalent types of extracellular vesicles (EVs) (1). They encapsulate a diverse array of bioactive constituents, such as proteins, lipids, nucleic acids, among others, and are abundantly found in saliva, blood, and various bodily fluids (2). Through engaging in intercellular communication among cancer cells, exosomes modulate cancer proliferation and migration (3). Wei et al. (4) demonstrated that OSCC cells inhibited the expression of Phosphatase and tensin homolog deleted on chromosome 10 by releasing exosomes carrying miR-130b-3p, which promoted OSCC tumor proliferation and angiogenesis. Guillaume (5) has demonstrated that proteins contained in extracellular vesicles, such as CD63, can reprogram target cells by altering cholesterol metabolism. However, there is still a lack of direct evidence regarding the precise regulatory impact of extracellular vesicles on cancer growth. The communication mechanism of extracellular vesicles needs further clarification, and their exact role in the early diagnosis and treatment of cancer deserves comprehensive investigation (6). Consequently, this review provides a succinct depiction of exosome structure, examines the mechanisms through which exosomes regulate cancers, and evaluates the advancements made in utilizing exosomes for the early detection and treatment of cancers.

## 2 Biological characteristics of exosomes

EVs secreted by cells can be broadly categorized into two types: ectosomes and exosomes. Ectosomes are vesicles released directly from the plasma membrane, ranging in diameter from 50 nm to 1 μm (7). EVs exhibit clear heterogeneity and can be roughly categorized into exosomes microvesicles and apoptotic bodies, with significant differences in size, function, and formation processes (8). Apoptotic bodies have the largest size, approximately 1000nm, and are vesicular structures containing DNA and cellular organelles formed during the process of cell apoptosis through cell membrane wrinkling and invagination (9). The diameter of microvesicles has a wide range of limitations and the mainstream view is that the diameter should be between 50-1000 nm (10). They are composed of an outer membrane with a lipid bilayer structure and various bioactive molecules, and play a role in disease diagnosis, cell communication, and microenvironment modulation (11). Exosomes are EVs with a smaller diameter and a microvesicle-like structure, which participate in cellular communication by carrying nucleic acids, proteins, metabolites, etc (12).

**Figure 1:**
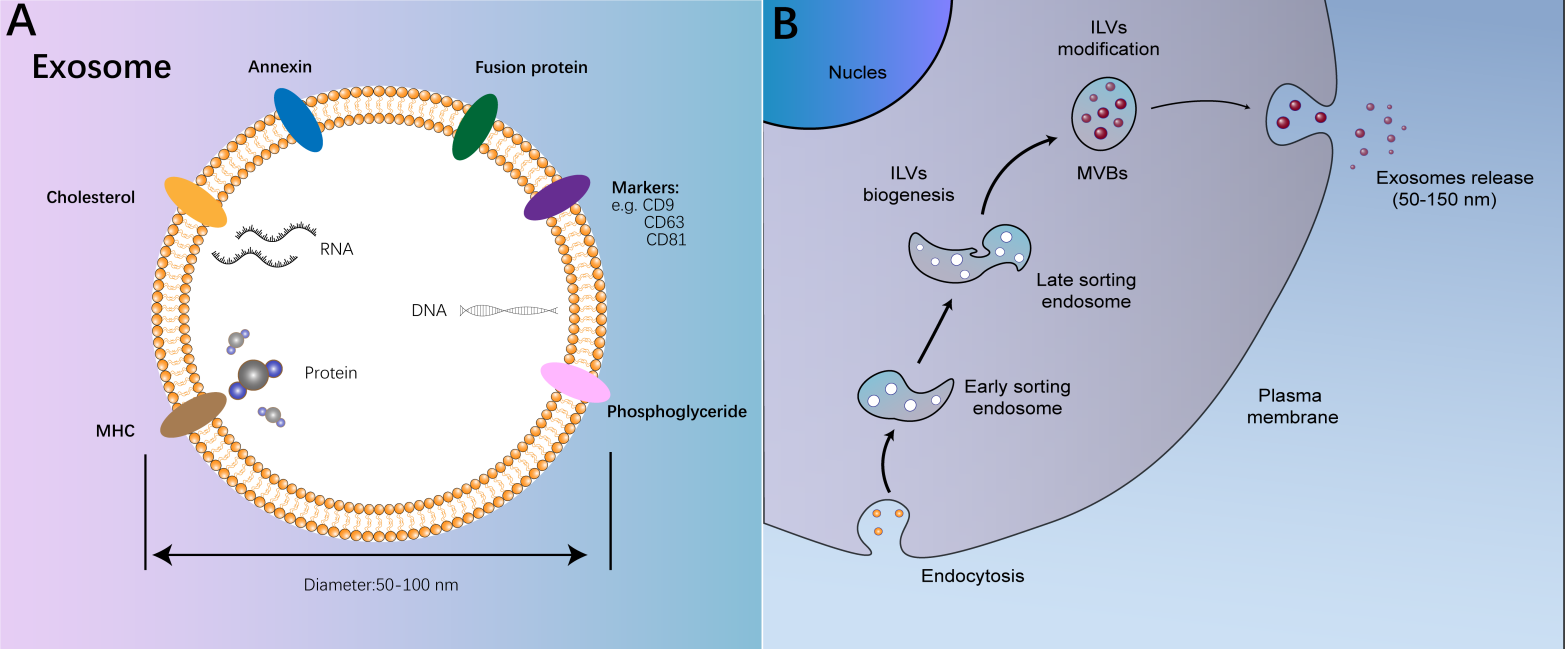
The structure and process on biogenesis of exosomes. **(A)** The structure and function of exosomes; **(B)** The process on biogenesis of exosomes.

This review centers on exosomes, which derive from the endosomal pathway and typically exhibit a narrow diameter range of approximately 40-160 nm (**Figure 1**) (13). Exosomes possess an outer layer consisting of a double lipid membrane and exhibit a density ranging from 1.13 to 1.19 g/ml (14). In an aqueous environment, they display an almost perfectly spherical structure; however, upon desiccation, they assume a distinctive cup-shaped morphology (15). Internally, exosomes are enriched with cholesterol and a diverse array of proteins (16). Initially characterized as cellular “garbage bags” replete with discarded cellular components, exosomes gained recognition in the 1990s for their role in B cell antigen presentation, prompting a reassessment of their significance in intercellular communication (17). Recent research has unveiled that cancer cells release exosomes containing cancer-specific molecules to engage in intercellular communication, a process intricately linked to cancer progression.

The movement of exosomes within the extracellular space plays a crucial role in mediating intercellular communication and is contingent upon the stability of the bilateral lipid layer structure of exosomes (18). Once exosomes are released into the extracellular milieu, only a fraction of their structure remains intact for a brief period (19). These exosomes have the capacity to promptly discharge bioactive molecules they encapsulate, including interleukin, transforming growth factor-β (TGF-β), vascular endothelial growth factor(VEGF), among others, which can bind to neighboring cells through specific receptors, facilitating intercellular communication (20). The outer membrane of most exosomes exhibits resistance to rupture due to the presence of various enzymatically active surface components acquired during intracellular modification (21). Consequently, these exosomes can traverse intercellular or bodily fluids to reach proximal or distal cells. This distinctive biological trait of exosomes implies their potential profound involvement in cancer progression.

## 3 Biogenesis and extraction of exosomes

### 3.1 Biogenesis of exosomes

The formation of intraluminal vesicles (ILVs) and multivesicular bodies (MVBs) constitutes a pivotal stage in exosome biogenesis, with the endosomal sorting complex required for transport (ESCRT) mechanism being the most crucial process in this pathway (22). The ESCRT machinery is a sophisticated assembly of approximately 20 proteins, organized into four distinct complexes: ESCRT-0, ESCRT-I, ESCRT-II, and ESCRT-III (23). ESCRT-0 functions as an endosomal cargo adaptor, playing a significant role in MVB formation initiation and serving as the trigger for the ESCRT cascade (24). While ESCRT-0 follows a nomenclature akin to other ESCRT complexes, it primarily kick-starts MVB formation without actively engaging in lipid bilayer restructuring, a topic not expounded upon here (25). The elongated structure of the ESCRT-I complex enables it to recruit the ESCRT-II complex (26). ESCRT-II, characterized by a Y-shaped configuration, features two Vps20 proteins at its apex, facilitating linkage to the underlying ESCRT-III complex (26). ESCRT-III, fundamental for its capacity to interact with negatively charged membrane components, is pivotal in mediating the separation of endosomal membranes and ILVs, serving as the core machinery in MVB formation (27). For an enumeration of the human ESCRT complex protein constituents (**Table 1**).

**Table 1:** Protein composition of the ESCRT complex in humans.

Complex	Protein composition
ESCRT-I	TSG 101
	VPS 28
	VPS 37A
	VPS 37B
	VPS 37C
	VPS 37D
	MVB 12A
	MVB 12B
	UBAP 1
	UBA 1L
	UMAD 1
ESCRT-II	EAP 30
	EAP 45
	EAP 20
ESCRT-III	CHMP 6
	CHMP 4A
	CHMP 4B
	CHMP 4C
	CHMP 3
	CHMP 2A
	CHMP 2B
	CHMP 1A
	CHMP 1B
	IST 1
	CHMP 7
	CHMP 5

ESCRT has been shown to be involved in exosome formation and functional regulation, and is closely related to the intracellular transport exosome release of Extracellular vesicles (MVEs), generally considered to be a monolayer of membrane-coated microvesicles containing multiple ILVs. Deborah (28) found that inhibition of the expression of ESCRT-III proteins Charged multivesicular body protein (CHMP)1A, CHMP1B, CHMP5 and IST1 homolog (IST1) could promote MVE cleavage and further inhibit Rab11a cell exosome activity by blocking MVE trafficking. Li et al. (29)provide that recombinant IQ motif Containing GTPase activating protein 1 (IQGAP1) protein can promote the release of IL-1β through exosomes and mediate intracelluar communication by bridging Gasdermin D (GSDMD) to the exosome’s ESCRT protein. However, currently there is still a lack of more systematic evidence regarding the mechanisms of ESCRT regulation in the occurrence and release of exosome (22). The role of ESCRT proteins in tumor invasion and migration in OSCC still requires further research.

### 3.2 Isolation of exosomes

Exosomes are ubiquitously present in the body and are accessible in various bodily fluids, including urine, serum, plasma, lymph, and cerebrospinal fluid (30). The isolation of exosomes marks the initial step in elucidating their functions; however, this process currently lacks standardized and universally accepted procedures (31). Presently, the most prevalent techniques for exosome isolation encompass ultracentrifugation, ultrafiltration, and immunocapture methods (32). The efficacy of ultracentrifugation in exosome isolation predominantly hinges on the exosome concentration within bodily fluids, yielding an overall efficiency ranging from 5% to 25% (33). Ultrafiltration, often employed in conjunction with ultracentrifugation, involves the use of a filter membrane with a diameter less than 800 nm to eliminate oversized vesicles, followed by a 200 nm filter membrane to capture exosomes (34). While this approach is simpler and more efficient, blockage of the filter membrane by a substantial number of particles can impede effective separation (35). Precipitation, a method that leverages the differential solubility of distinct exosome types to isolate specific exosomes, offers high efficiency and rapidity but may compromise the structural integrity of exosomes (35, 36).

In the Minimal information for studies of extracellular vesicles (MISEV2023) published by the International Society for Extracellular Vesicles (ISEV) on the topic “From basic to advanced approaches”, which stated that differential ultracentrifugation (dUC) is still the most mainstream exosome extraction method (37). Unfortunately, dUC alone cannot perfectly isolate exosomes, and further separation/concentration is carried out by relying on the biophysical properties of exosomes such as size, density, charge, and surface composition. Size exclusion chromatography (SEC) is a commercially available, highly reproducible method for exosome separation based on differences in molecular size and matrix elution rate (38). Its separation efficiency is influenced by various factors such as the chromatography column, buffer solution, and pressure (39). ImmunoAffinity Capture is an exosome separation method based on ‘Affinity Separation’ technology, the basic principle of which is to use the Pull down/flow away mechanism of the screening matrix to concentrate the exosome by binding the specific immune probe to the surface protein of the exosome (40). The stable and mature exosome separation technology has laid a solid foundation for its clinical diagnosis and therapeutic applications.

## 4 Research progress on the biological role of exosome in OSCC

The biological functionality of exosomes predominantly relies on the transportation of their bioactive contents. In this section, we will elaborate on the various biological roles of distinct exosome types in Oral Squamous Cell Carcinoma (OSCC) based on their diverse contents.

### 4.1 Exosome regulates OSCC growth by transporting growth factors

Exosomes promote cancer progression by transporting various growth factors, such as epidermal growth factor (EGF), VEGF, and fibroblast growth factor (FGF), to the tumor microenvironment (41). These growth factors trigger the proliferation and metastasis of cancer cells, leading to tumor enlargement. Angiogenesis, the development of new blood vessels, is a pivotal process crucial for cancer progression and metastasis (42). The research conducted by the Liu team (43) indicates that VEGF secreted by OSCC can bind to heparan sulfate proteoglycans (HSPG) proteins on exosomes, thereby increasing local levels of heparan sulfate proteoglycans (HSPG) and promoting OSCC growth. Zeng et al.’s (44) research has demonstrated that TGF-β can inhibit Smad4 nuclear translocation and promote the metastasis and migration of OSCC tumors. The PDFG-containing exosomes released by OSCC can up-regulate the expression of MiR-3529-3p by regulating cancer-associated fibroblasts (CAFs), and promote the proliferation of OSCC with positive feedback (45). Exosomes released by OSCC cells possess the ability to promote angiogenesis by transporting pro-angiogenic factors such as VEGF, platelet-derived growth factor (PDGF), and transforming growth factor-beta (TGF-β) to neighboring endothelial cells (46). These factors induce the formation of new blood vessels, supplying essential nutrients and oxygen to the OSCC, thereby enhancing its progression (47).

### 4.2 Exosome regulates OSCC growth by transporting nucleic acids

Exosomes have the capacity to transport a plethora of RNA molecules, encompassing mRNA, miRNA, and ncRNA, actively participating in the regulation of cancer growth and migration (48). Cintia et al. (49) found that the dysregulation of miR-497-5p and miR-4417 in OSCC promotes growth and proliferation through the regulation of the Chondroitin sulfate/Dermatan Sulfate (CSPG/DSPG) and Keratan Sulfate (KSPG) proteoglycans molecular pathway. Although exosomal miRNAs have been shown to have regulatory effects in a variety of tumors, more evidence is needed on the role and mechanism of exosomal miRNAs in the growth, migration, and mesenization of OSCC (49, 50).

Noncoding RNA (ncRNA), a prevalent RNA type found in exosomes, comprises short RNA molecules transcribed from DNA that do not encode proteins (51). In their research, Du et al. demonstrated that exosomes containing long noncoding RNA (lncRNA) extracted from OSCC culture substrates could impede the proliferation and migration of OSCC by modulating the miR-17-5p/SOCS6 axis in OSCC (52).

Current findings suggest that neighboring cells of cancer can influence the growth and migration of OSCC by releasing RNA-loaded exosomes (53). Wang et al. (54) discovered that Cancer-Associated Fibroblasts (CAFs) release exosomes containing mRNA, which suppress immune responses and boost OSCC proliferation through mRNA-miRNA interactions.

These observations underscore the crucial role of cancer cell-released exosomes in fostering cancer development, angiogenesis, invasion, and metastasis (55). Hence, deciphering the mechanisms by which exosomes impact cancer progression could potentially pave the way for innovative cancer treatment strategies.

## 5 Applications of exosomes in OSCC diagnosis

The detection of exosomes in OSCC harbors substantial potential in cancer diagnosis. In a systematic analysis conducted by Sahu et al. (56), it was concluded that various biomarkers including human papillomavirus type 16 protein E7 (HPV-16-E7), Mucin 16, signal-regulatory-protein-α (SIRPA), annexin A1, phosphorylated epidermal growth factor receptor antibody (phospho-EGFR) and heat shock protein 90 (HSP90) serve as promising early diagnostic indicators for OSCC, offering significant enhancements in patient prognosis. Kisho et.al (57) has found that knocking down HSP90 can effectively inhibit lymphatic metastasis in OSCC, and detecting HSP90 can effectively determine the presence of lymphatic metastasis in OSCC. The transmembrane glycoprotein SIRPA can induce heterotrimeric G protein signaling by interacting with integrins of the β1, β2, and β3 families, promoting the evasion of OSCC from macrophage phagocytosis, and has the potential to be used as a diagnostic marker (58). Utilizing body fluid biopsies to analyze exosome contents like miRNA, mRNA, and ncRNA for OSCC screening presents advantages such as cost reduction, minimal invasiveness, and early detection (59).

The composition of exosomes in the cancer microenvironment exhibited significant variations across distinct stages of OSCC (60). Investigating the exosome content profiles in OSCC, the disease progression statuses, and the potential targeted therapeutic approaches holds theoretical promise. Regrettably, owing to the current deficiency in detection techniques, the limited understanding of exosome subtypes, and the incomplete comprehension of OSCC pathogenesis, this framework remains purely theoretical, necessitating further empirical validation.

Prognostic assessment is another important application of exosome testing in diagnosis. Detecting the contents of exosomes in OSCC also aids in assessing the prognosis of cancer patients (61). Chen et al. (62) observed that up-regulation of miRNA-155 and miRNA-21 in exosomes corresponded with a significant down-regulation of gene of phosphate and tension homology deleted on chromosome ten (PTEN) and B-cell CLL/lymphoma 6 (Bcl-6) expression in OSCC, indicating heightened proliferative activity and invasion potential in the cancer. MiR-21, which is transported by exosomes, can target PTEN and programmed cell death 4 (PDCD4) proteins by reprogramming the NF-κB pathway in OSCC cells, conferring cisplatin-resistance in OSCC cells (63). Patients with high miR-21 expression may have a less favorable prognosis. Pang et al. (64) demonstrated that elevated levels of CKLF-like MARVEL transmembrane domain-containing 6 in exosomes were associated with a notably poorer prognosis in OSCC.

Some of the exosome tumor diagnosis tests have entered the clinical trial stage. Krug utilizes the changes in exoRNA from the peripheral exosomes to diagnose nonsmall-cell-lung-cancer with a sensitivity of 98% using epidermal growth factor receptor (EGFR) mutations (65). Lee (66) extracted extracellular vesicles from 310 urine samples and amplified the extracellular vesicle miRNA cDCA library using RT-PCR and qPCR were used to measure the content of bkv-miR-B1-5p, achieving positive results in the assessment of immunologic risk and tolerance in kidney transplantation (ARKTK). However, clinical trials using exosomes and related assays for the diagnosis of OSCC have not been reported.

Currently, utilizing exosomes for diagnosing OSCC offers several advantages over traditional pathological biopsy methods: (i) Exosome-based diagnosis requires only a small sample, enabling the assessment of OSCC with minimal specimen sizes following a straightforward extraction and purification process (67); (ii) Exosomes exhibit high sensitivity, directly interacting with cancer cells to provide immediate feedback on the current state of OSCC development (61); (iii) Advancements in detection technologies and theoretical understanding enhance the potential application of exosome-based diagnostics for early detection and grading of OSCC (68). The team led by LEE (66)has successfully utilized urinary exosomes to detect whether the BK virus (BKV) has recurred, thereby assessing the risk of tumors after kidney transplantation. This demonstrates the potential clinical application of exosomes. Despite the numerous benefits of exosome-based OSCC diagnosis, its diagnostic accuracy still lags behind that of tissue biopsy, conventionally regarded as the gold standard for OSCC diagnosis (59). Cheng et al. (69) found in diagnostic testing for high-risk ground-glass opacities in the lungs that exosome testing detected 9 out of 10 cases, while immunohistochemistry achieved a detection rate of 100%. The reason for the problem is insufficient technology in the extraction, classification, and purification of exosomes (70). Microfluidic-based exosome isolation technology uses an automated centrifugal microfluidic disc system with functionalized membranes to concentrate and collect exosomes in as rapid as 8 minutes (71). Unfortunately, the integration of the concentration, purification, and identification steps in this plan has not yet been achieved. Further research and evidence are still required for clinical translation (71). Unfortunately, there have been no reported specific methods for isolating OSCC tumor-derived extracellular vesicles at present.

In conclusion, further refinement of detection techniques is essential for the diagnostic strategy involving exosome-related OSCC, along with the accumulation of additional clinical evidence to support these methods. These investigations remain focal areas in the field of exosome-related OSCC diagnosis.

## 6 Application of exosome in the treatment of OSCC

Exosomes possess significant potential for cancer therapy owing to their excellent biosafety profile and effective delivery capabilities. In the realm of cancer treatment, exosomes can be categorized into artificially modified exosomes and naive exosomes based on their characteristics (72). Naive exosomes, extracted unaltered from cells *in vivo*, harbor bioactive molecules from their cellular source and demonstrate therapeutic efficacy accordingly (73). Modified exosomes are typically generated through two primary methods: (i) Genetic engineering techniques are employed to induce low or high expression of specific proteins/nucleic acids in secretory cells, yielding modified exosomes (74). (ii) Exogenous nucleic acids or nanoparticles are introduced into exosomes via processes such as electroporation, liposome transfection, or ultrasound (75). Modified exosomes, in contrast to naive exosomes, exhibit enhanced therapeutic potential due to their richer cargo of bioactive molecules (72).

Currently, several clinical trials investigating exosome-based therapeutics are in progress. For instance, mesenchymal stem cell-derived exosomes are being explored for the treatment of conditions such as diabetes mellitus (NCT02138331), lymphoma (NCT04223622), osteoarthritis (NCT04223622), Alzheimer’s disease (NCT04388982), and ischemic stroke (NCT02458755) (62, 76). Despite these advancements, the efficacy of exosome-based drugs in managing OSCC remains uncertain. This review aims to delve into the effects and underlying mechanisms of exosome-related therapeutics in OSCC treatment.

### 6.1 Naïve exosome on OSCC treatment

Research has illustrated that the fusion of naive exosomes discharged by immune cells, notably NK cells, with cancer cells can facilitate cancer cell apoptosis (77). Nikfarjam et al. (77) documented that exosomes emitted by dendritic cells exhibit a substantial capacity to impede the proliferation of epithelial-derived cancer cells. While extensive evidence supports the inhibitory effects of naive exosomes on the proliferation and invasion of epithelial-derived cancers, further substantiation is required to elucidate the therapeutic efficacy and underlying mechanisms of naive exosomes in the context of OSCC.

### 6.2 Modified exosome on OSCC treatment

In comparison to naive exosomes, modified exosomes offer the benefits of enhanced targeting, heightened efficacy, and simplified operation, making them increasingly preferred by researchers (78).

Exosomes can enhance the specificity of cancer cell targeting and facilitate precise delivery by tailoring specific proteins or molecules on their surface. For instance, the bone-targeting protein WYRGRL has been engineered onto exosome surfaces to precisely direct them towards bone tissue for addressing joint inflammation (79). Zhou et al. (68) have also engineered exosome surfaces with immunogenic cell death triggers, indicating their potential for targeting pancreatic cancer to bolster therapeutic outcomes. Some researchers have developed pH-responsive exosomes that capitalize on the acidity of the cancer microenvironment, enabling rapid breakdown within acidic conditions to achieve accurate drug delivery (80). Building on these approaches, Kase et al. integrated genetic engineering and electroporation techniques to craft OSCC-targeting exosomes containing siLCP1, effectively impeding OSCC progression (81). Enhancing the specificity of exosome targeting toward OSCC cells remains a focal point within exosome-based cancer therapy research.

The exceptional biological safety and targeting attributes of exosomes for efficient drug conveyance continue to underpin the treatment paradigm for OSCC with exosomes. Li et al. (82) loaded photosensitive nanoparticles, such as Indocyanine green, into exosomes, delivering them to OSCC cells to induce potent cellular damage and impede OSCC advancement. Huang et al. (83)engineered exosomes with heightened expression of miR-144/451a via transfection. Leveraging the synergistic regulatory effects of miR-144/451a on OSCC cell invasion and proliferation, a notable reduction in the survival, migration, and invasion capabilities of OSCC cells was observed. Sayyed et al. (84) pursued an exosome adjuvant therapy strategy utilizing exosomes enriched with miR-155 to counteract OSCC resistance to cisplatin, thus enhancing the efficacy of chemotherapy agents. Collectively, these findings underscore the substantial advantages of exosomes in precision drug delivery for OSCC treatment.

Nonetheless, the mechanisms governing drug delivery into OSCC cells remain elusive. Effectively regulating local drug release and mitigating drug-related side effects present ongoing challenges, warranting further investigation.

## 7 Conclusions and challenges of exosomes on OSCC diagnosis and treatment

Although the potential application of exosomes in OSCC diagnosis and treatment holds promise, there are several associated challenges. One such challenge pertains to isolation and purification of exosomes. Being minute, membrane-bound vesicles that can be difficult to isolate and purify from other EVs and contaminants in biological fluids. The methods employed for isolation and purification can significantly impact the purity, and quality of exosomes, which affects their therapeutic efficacy. Therefore, standardized isolation and purification protocols need to be developed to ensure consistent and reproducible results (33).

Another challenge is the scalability of exosome production. Exosomes are typically produced in small quantities, and the process of isolating and purifying exosomes can be time-consuming and costly. To facilitate the clinical utilization of exosomes, it is crucial to devise scalable production techniques capable of generating substantial quantities of exosomes cost-effectively.

Furthermore, the safety of exosome-based therapy necessitates thorough evaluation. While exosomes have exhibited good tolerance in animal trials, the enduring impacts of exosome therapy on human well-being remain inadequately elucidated. Moreover, the probability of off-target repercussions and inadvertent outcomes stemming from exosome treatment demands meticulous scrutiny.

Finally, the regulatory framework for exosome-based therapies in OSCC is still evolving. Exosomes are considered biological products by regulatory agencies, such as the U.S. Food and Drug Administration (FDA), and their clinical use is subject to regulation and approval. Therefore, establishing a regulatory pathway for the development and approval of exosome-based therapies in dental medicine is important.

In general, challenges related to the utilization of exosomes in OSCC diagnosis and treatment encompass isolation and purification, scalability, safety, and regulatory aspects. Effectively tackling these challenges will be vital in unlocking the complete therapeutic potential of exosomes within the realm of dental medicine.
